# Biotin induced biochemical hyperthyroidism: a case report and review of the literature

**DOI:** 10.1186/s13256-023-04002-z

**Published:** 2023-06-28

**Authors:** Archa James, Jinu Stalan, Jose Kuzhively

**Affiliations:** 1 Government Medical College, Kerala University of Health Sciences (KUHS), Thiruvananthapuram, India; 2 Department of Endocrinology, Diabetes and Metabolism, UnityPoint Clinic, UnityPoint Health-Methodist Hospital, Peoria, IL USA

**Keywords:** Biotin, Biotin/streptavidin immunoassay, Biotin interference, Biochemical hyperthyroidism, Misdiagnosis, Awareness

## Abstract

**Background:**

Biotin is a commonly used supplement for hair, nail, and skin. Recent literature suggests that high-dose biotin therapy for neurological diseases like Multiple sclerosis can interfere with lab results that use biotin/streptavidin immunoassay, called biotin interference. Biotin interference can affect thyroid lab results, giving biochemical hyperthyroidism.

**Case presentation:**

Our patient, a 64-year-old white man with a known history of multiple sclerosis, presented with elevated free T3, free T4, and low TSH that resembled hyperthyroidism. He had no symptoms of hyperthyroidism except some fatigue and tachycardia on the first encounter. He was started on anti-thyroid medications. He was then re-evaluated since his lab results remained the same after two months of anti-thyroid medications. It was found that he was on biotin, 10000mcg/day, for his multiple sclerosis. Biotin was discontinued, and five days later his lab results returned to normal values.

**Conclusion:**

The lack of knowledge of biotin use by patients can lead to misdiagnosis of patients’ thyroid lab results and improper management. Awareness about biotin interference and abnormal thyroid lab values should be a priority among clinicians and the public. If the biotin is discontinued on time, such misdiagnosis can be avoided.

## Background

Biotin, (B7) is a water-soluble vitamin found in egg yolks, legumes, nuts, liver, and so on. It is a readily available supplement over-the-counter in the names of vitamin B7, Vitamin H, and Coenzyme R and may be a component of other supplements. Biotin is an essential cofactor for five carboxylases involved in the fatty acid synthesis and energy production. Biotin deficiency is rare and the recommended daily intake (RDI) ranges from 30 to 70 mcg per day [1]. Biotin is good for eyes, hair, nails, skin, and brain function. At high doses (10,000 times RDI), biotin may slow the advancement of progressive multiple sclerosis, and improves clinical outcomes and quality of life in patients. It is also prescribed for several other rare metabolic diseases like biotinidase deficiency and biotin-thiamine-responsive basal ganglia disease (BTBGD). Supra-physiological doses of biotin are used for self-medication for hair loss [1]. The potential medical issue is that supra-physiological biotin intakes can cause spurious blood results in streptavidin/ biotin-based immunoassays used for routine clinical laboratory tests, such as thyroid, steroid, polypeptide hormones, tumor markers, and vitamins [4]. Thyroid bioassay interference may fully mimic and is indistinguishable from the typical biochemical picture of Grave’s disease and sometimes persist for several days after biotin withdrawal [3].

## Case presentation

Our patient is a 64-year-old white male with a known history of Multiple sclerosis, Obstructive Sleep Apnea, Chronic Obstructive Pulmonary Disease, Benign Prostatic Hyperplasia, and Dyslipidemia. He was on lipid lowering therapy, tadalafil, tamsulosin, Pramipexole and other Over-the-Counter medications.He used to work for a production company.

He was referred by his primary care physician to our office in light of abnormal thyroid values suggestive of hyperthyroidism. His thyroid ultrasound was unremarkable. He had symptoms of fatigue and hair loss. His weight loss was intentional. On examination, his vitals were normal except tachycardia. He had a Body Mass Index of 38.41. His systemic examination was irrelevant and had a normal thyroid. He was advised to repeat the thyroid function test, the Thyroid Stimulating Immunoglobulin assay and the Thyroid Peroxidase Antibody test. His lab results returned with low TSH, elevated free T3 and T4 with negative antibody screening. All other blood and urine results were normal. Apathetic hyperthyroidism in the elderly can present with fatigue, so he was suspected of grave’s disease and started on Methimazole, 40 mg, daily and Metoprolol, 50 mg, daily. One month later, he was euthyroid, with no symptoms or signs of hyperthyroidism, but the labs showed an increase in Free T4 (Ft4) and Free T3 (FT3) levels. Therefore, his medications were changed to Propylthiouracil, 150 mg, three times, daily. One month later, he had similar lab values with low TSH and elevated T3 and T4. Since the underlying etiology for his non-responsiveness to medications could not be found, and given the possible medication resistance he was advised to take the nuclear uptake scan. The scan showed homogenous activity in the thyroid gland with no hot/cold nodules. Given the regular study, biochemical hyperthyroidism, non-responsiveness to medications, and clinically euthyroid, his medical history was evaluated in detail and found that he was on high dose biotin, 10,000 mcg daily for his Multiple sclerosis. He was advised to stop biotin and repeat the thyroid labs after five days. The result returned to normal. His anti-thyroid medications were discontinued. The labs repeated one month later were normal. He was advised to stop taking biotin 3 or 4 days before thyroid lab tests.

The Thyroid lab results of our patient are shown in Table 1.

**Table 1:** Test results

	06/25/2018 off biotin	05/23/2018 off biotin	05/10/2018 on biotin	04/20/2018 on biotin	03/07/2018 on biotin	Reference range
TSH	1.66	1.58	0.03	0.04	0.06	0.4–4.0 µg/dl
Free T4	0.86	0.77	> 8.00	5.17	4.82	0.9–1.7 ng/dl
Free T3	2.9	3.8	> 30.0	> 30.0	> 30.0	2.3–4.1 pg/ml
TSI index					1.01	< 1.30
TPO Ab					< 28	< 34

*TSI index* Thyroid Stimulating Immunoglobulin Index

*TPO Ab* Thyroid peroxidase Antibody

## Discussion

Biotin is a water-soluble B vitamin that is plentiful in both plants and animal foods such that biotin deficiency is extremely rare [5]. It is an essential coenzyme involved in the CO2 transfer in carboxylation reactions including, gluconeogenesis, lipogenesis, and fatty acid synthesis. The recommended daily intake of biotin is 30 µg/d [4]. In addition to its presence in multivitamin preparations, it has become commonly used as a supplement for skin, nail, and hair health [5].

Generic formulations of biotin up to 10 mg are widely available over-the-counter [3]. Several rare, inherited metabolic diseases can benefit from the High Dose of Biotin (eg: biotinidase deficiency: 5–10 mg/day; holocarboxylase synthetase deficiency: 30–40 mg/day; biotin-thiamine-responsive basal ganglia disease: 100-300 mg/day) [3]. Doses up to 30 mg/day are now widely used for improving hair, nail, or skin conditions and for supportive treatment in mitochondrial energy metabolism disorders, lipid disorders, and diabetic peripheral neuropathy [3]. The mega doses of biotin (100-300 mg/d) apply to patients with primary or secondary progressive multiple sclerosis in which clinical outcomes have improved by using biotin as a complementary medicine [4].

Biotin is generally believed to have favorable tolerability and safety to individuals, even when individuals have received pharmacological doses up to 300 mg/d [4]. The potential medical issue is that supra-physiologic biotin intakes can cause spurious results in streptavidin/biotin-based immunoassays used for routine clinical laboratory tests [4]. While the amount of biotin naturally received through dietary means is not enough to interfere with clinical tests, the levels found in these supplements can generate significant errors [2].

Biotin is small and thus able to bind to a wide variety of molecules without altering their chemical properties [2]. The streptavidin/biotin complex represents one of the most robust non-covalent interactions in nature. This complex is not disturbed by multiple washing steps, and biotinylation typically does not alter the biological activity or immunologic specificity when bound to test molecules [3].

Biotin mainly interferes with two types of clinical assays: Sandwich assay and competitive assay. Biotin interference causes falsely high results with competitive immunoassays used to measure small molecules (free T4, free T3, total T4, Total T3, cortisol, 25- hydroxy vitamin D) and causes falsely low results with sandwich assays used to measure large molecules (TSH, β- hCG, PTH, insulin, ferritin, pro-b-type natriuretic peptide, prostate-specific antigen, and others [5, 6].

As influenced by high biotin levels, thyroid function test results could mimic hyperthyroidism with low TSH, high TT3, high fT3, and high fT4 [5]. In addition, biotin also interferes with the detection of anti-thyrotropin antibodies, so it can result in a laboratory pattern identical to Graves’ disease [6]. There have been a large number of reports concerning misdiagnosed Graves’s disease due to biotin interference. Second, falsely elevated free T3 and free T4 levels associated with normal TSH levels may be misdiagnosed as rare causes such as central hyperthyroidism or thyroid hormone resistance, resulting in unnecessary interventions.

Various factors may contribute to the artificially falsely high or low results, including the degree of blood biotin elevation based in turn on the amount of biotin ingested, the time interval from biotin ingestion to blood specimen collection, the biotin interference threshold, and the patient’s own relative biotin metabolism [5]. It is important to note that high levels of biotin supplementation strictly interfere with in -vitro lab tests. The patients’ abnormal blood results would not be reflected in the patient, who is likely to be asymptomatic. Therefore, it is crucial to maintain a high index of suspicion and ask about biotin supplementation levels when an otherwise asymptomatic patient presents with abnormal results [2].

Our patient, who was taking 10000mcg/day biotin for Multiple Sclerosis showed lab results of falsely elevated free T3 and T4 and falsely low TSH. His thyroid function report returned to normal when he stopped taking biotin for five days. Even though similar cases are reported, this abnormal lab test is still underdiagnosed. Through this case report we reinforce the importance of being aware about the conditions where biotin is used and the interference it can cause with thyroid labs.

Biotin remains an under-recognized cause of abnormal lab results. These lab errors can cause emotional strain on the patient, lead to costly and unnecessary work-ups, and potentially harmful and unnecessary interventions [2]. It is worthwhile to rule out biotin supplementation before diagnosing abnormal thyroid function (especially before irreversible treatments such as radioactive iodine ablation). Given the increasingly common use of biotin supplements in high dosage and the prevalence of hypothyroidism with dependence on periodic measurement of thyroid function tests for adjustment of T4 dosage, there is significant potential for clinical mismanagement of these patients based on misleading test results. The same concern applies to patients on levothyroxine post-thyroidectomy or patients with thyroid hyperfunction treated with radioiodine or anti-thyroid drugs. Determination of biotin intake would be particularly important in situations requiring more exact titration of levothyroxine dosage, such as in pregnant women, children, the elderly, and in patients monitored for residual or recurrent thyroid cancer [5].

The U.S. Food and Drug Administration (FDA) released safety communication to remind the public, healthcare providers, lab personnel, and lab test developers that biotin, often found in dietary supplements, can significantly interfere with certain lab tests and cause incorrect results that may go undetected [7]. If a patient insists on taking high dose biotin, they may do so without affecting thyroid function tests if they hold it long enough before doing the labs [6]. The biotin should be discontinued for at least 48–72 h before thyroid assaying by any commonly used biotin-streptavidin immunoassay [2].

## Conclusion

Biotin interference can cause many lab test abnormalities, including thyroid function tests. Thyroid lab results resemble hyperthyroidism and may lead to a misdiagnosis and potentially serious clinical implications. Clinicians, Nutritionists, physicians, nurses, and other healthcare providers should include biotin in the nutritional history of patient groups that might have instituted biotin therapy, such as Multiple Sclerosis. Patients do not always recognize the name biotin, so it is prudent to ask if the patient is taking any supplements for hair, nails, or skin. The biotin should be discontinued for at least 2–3 days before thyroid assays using biotin/streptavidin immunoassay.

## Data Availability

Not applicable.
